# Thyroid-Related Hormone Levels in Clinical Patients With Moderately Severe-to-Profound Sudden Sensorineural Hearing Loss: A Prospective Study

**DOI:** 10.3389/fneur.2021.753270

**Published:** 2021-10-28

**Authors:** Zhong Zheng, Ying Shen, Liang Xia, Lili Xiao, Yuanyuan Sun, Hui Wang, Zhengnong Chen, Yaqin Wu, Haibo Shi, Jingchun He, Yanmei Feng, Shankai Yin

**Affiliations:** ^1^Department of Otolaryngology, Shanghai Jiao Tong University Affiliated Sixth People's Hospital, Shanghai, China; ^2^Shanghai Key Laboratory of Sleep Disordered Breathing, Shanghai, China; ^3^Department of Otolaryngology, Shanghai Jiao Tong University Affiliated Xinhua Hospital, Shanghai, China

**Keywords:** moderately severe-to-profound sudden sensorineural hearing loss, thyroid-related hormone, predictor, triiodothyronine, thyroid stimulating hormone

## Abstract

**Objectives:** Sudden sensorineural hearing loss (SSNHL) is a common otological emergency, causing a measure of hearing loss and affecting the quality of life. This study aims to investigate the association of thyroid-related hormone levels with moderately severe-to-profound SSNHL.

**Methods:** The study included 70 patients with moderately severe-to-profound SSNHL and 100 age- and sex-matched healthy controls. Peripheral venous blood samples were taken from the participants, and their thyroid-related hormone levels were measured at admission and 1 week after treatment.

**Results:** In moderately severe-to-profound SSNaHL patients, the concentrations of total triiodothyronine (TT3), total thyroxine (TT4), free triiodothyronine (FT3), and thyroid-stimulating hormone (TSH) (all *P* < 0.05) were significantly lower than in the control group. The TT3, TT4, FT3, and TSH levels were significantly higher in the effective group than in the ineffective group (all *P* < 0.05). Linear correlation analysis revealed that TSH level (*R* = 0.707, *P* < 0.05) elevation after treatment successfully predicted a favorable outcome of hearing recovery. Logistic regression analyses suggested low FT3 and TSH levels to be independent occurrence predictors, while the increase of TSH level may be an independent favorable outcome predictor.

**Conclusions:** The results suggest that low FT3 and TSH levels are risk factors for moderately severe-to-profound SSNHL. By discovering the positive association between TSH elevation and hearing recovery, along with the potential novel predictors of FT3 and TSH, our study may contribute valuable insights to the research and treatment of moderately severe-to-profound SSNHL.

## Introduction

Sudden sensorineural hearing loss (SSNHL) is defined as a rapid onset of hearing impairment with more than 30 dB decrease in at least three continuous frequencies within 72 h (1). Although the morbidity rate of SSNHL in China has been increasing in recent years, large-scale epidemiological data are still lacking. Referring to data from the United States, the incidence of SSNHL ranges from 5 to 27 per 100,000 population, with about 66,000 new cases per year (2–4). SSNHL usually occurs unilaterally, and is sometimes accompanied by tinnitus, vertigo, ear fullness, and nausea. It is a particularly devastating disease mainly due to a lack of understanding about its causes, exacerbated by delays and limited options for treatment (5). Hearing loss frequency and degree, age, presence of vertigo, and the initial time of therapy are all factors that may influence the prognosis of SSNHL (6). Among these factors, the degree of hearing loss plays the most important role (7). Sheehy et al. (8) reported that primary hearing loss with a decrease in intensity of 45 dB or lower presented satisfactory recovery prognosis. In contrast, Enache et al. (9) reported that hearing loss with a decrease in frequency of more than 50–60 dB, even with adequate treatment, leads to a recovery of no more than 20–30 dB. Steroids that are effective for most cases have no effect when the initial hearing loss is 90 dB or greater (10). Generally, high hearing loss severity translates to a worse prognosis. According to the latest World Health Organization hearing classification, hearing loss of more than 50 dB is classified as moderate to severe deafness, where patients experience difficulty in hearing conversational speech (11). Thus, our research focused on moderately severe-to-profound SSNHL at all frequencies.

The guidelines of China (2015) (6) emphasize that moderately severe-to-profound SSNHL at all frequencies may be caused by vasospasms or endothelial dysfunction, and inner ear embolism or thrombosis. Thyroid disorders are risk factors for cardiovascular and cerebrovascular disease, including acute ischemic stroke, atherosclerosis, and myocardial infarction (12–14). Hypothyroidism has a significant causal association with a worse profile of atherosclerotic risk factors and may play a role in atherothrombotic myocardial infarction (15). Given the similar pathogenesis of cardiovascular disease, cerebrovascular disease, and moderately severe-to-profound SSNHL, we hypothesize that comparing the thyroid function tests conducted upon admission and after treatment may assist with the diagnosis and prognosis of moderately severe-to-profound SSNHL. These cover measurements of total triiodothyronine (TT3), total thyroxine (TT4), free triiodothyronine (FT3), free thyroxine (FT4), and thyroid stimulating hormone (TSH). Thyroid hormones play vital roles in cochlear development and in the maintenance of adulthood hearing (16, 17). Ng et al. (18) and Forrest et al. (19) demonstrated that thyroid hormones and their receptors are required for the development of hearing. Richter et al. (20) and Ng et al. (21) supported the same concept reporting that T3 is essential for normal cochlear function and morphology in mice, where a lack of T3 could lead to important alterations in cochlear morphology and loss of cochlear function. T3 regulates not only the development of auditory function but also the maturation of auditory sensitivity, which is evident through reports from Li et al. (22) who reported hypothyroidism to be associated with sensorineural hearing loss.

To investigate the potential roles that thyroid-related hormone may play in the development of moderately severe-to-profound SSNHL, we designed this prospective study to compare the thyroid hormone levels before and after treatment for the first time. A regression analysis was done to determine the occurrence and prognosis, and linear regression was performed to compare thyroid-related hormone vs. the severity of hearing loss, and the elevation of thyroid-related hormone vs. hearing recovery. Receiver operating characteristic (ROC) curve analysis was used to assess the predictive value of thyroid-related hormone for SSNHL.

## Materials and Methods

### Study Population

A total of 70 consecutive patients with moderately severe-to-profound SSNHL diagnosed at our hospital between July 2018 and December 2020 were prospectively enrolled. All participants provided written informed consent for their inclusion in the database and the use of their data for research purposes. The study protocol was approved and implemented according to the ethical standards of the Institutional Ethics Committee of the Shanghai Jiao Tong University Affiliated Sixth People's Hospital [2018-KY-036(K)]. The progress was conducted in accordance with the spirit of the Helsinki Declaration. Patients included in the study visited the hospital for the first time within 7 days after the onset of moderately severe-to-profound SSNHL. All participants underwent standard laboratory tests and audiological diagnostic procedures. Included patients were those with hearing loss at all frequencies and mean pure tone audiometry (PTA) across 0.25–8 kHz ≥50 dB. Those excluded had acute inflammatory conditions, obstructive sleep apnea, connective tissue diseases, abnormal ear examination findings, a previous history of thyroid disorder, chronic otitis media, a history of acoustic trauma or otologic surgery, conductive hearing loss, or had used ototoxic medications. Patients with malignant disease, psychiatric conditions, dementia, hepatitis B or C, or other major comorbidities (heart failure; stroke; and severe hepatic, pulmonary, or renal dysfunction) were also excluded. A group of 100 sex- and age-matched controls without any disease at regular health check-ups was used for comparison. The exclusion criteria were the same as those for the SSNHL group.

### Data Collection

The baseline characteristics included age, sex, height, weight, body mass index (BMI), and blood pressure on admission (systolic blood pressure and diastolic blood pressure). The clinical characteristics included affected side, accompanying symptoms (including tinnitus, vertigo, ear fullness), history of hypertension and diabetes, time to treatment, and hearing level on admission. All hearing assessments were performed in standard shielding rooms and PTA was performed for both air and bone conduction at 0.125, 0.25, 0.5, 1, 2, 4, and 8 kHz before and after 7 days' course of systemic treatment. The hearing loss of each individual was calculated by averaging the PTA value of damaged frequencies after onset. The extent of hearing recovery is calculated using PTA after onset minus PTA after treatment. All patients underwent temporal bone computed tomography or inner ear magnetic resonance imaging, ensuring no ear structural abnormality and tumors were found. Upon admission, thyroid function tests of TT3, TT4, FT3, FT4, and TSH levels were performed using blood samples obtained from the antecubital veins of all patients between 6 and 7 a.m. after an overnight fast. The thyroid function test was performed again post-treatment and its difference (post-minus pre) from the first test was used to evaluate the changes in thyroid-related hormone.

### Treatment Procedure

Once SSNHL was diagnosed, patients were hospitalized for 1 week. All the patients underwent comprehensive treatment, including treatment with steroids and batroxobin following the 2015 China guideline for the diagnosis and treatment of sudden deafness (6). The comprehensive treatment consisted of intravenous injection of Prednisone (1 mg/kg/day) for 3–5 days, followed by a reduced dosage for the remaining days according to the hearing improvement, and intravenous batroxobin (10U batroxobin for the first time and then reduced to 5U batroxobin, once every other day, 1–3 times in total according to the level of fibrinogen). Patients that experienced hearing recovery were divided into the effective group (PTA of impaired frequencies which improved more than or equal to 15 dB, or back to normal/unaffected ear) and the ineffective group (PTA of impaired frequencies which improved <15 dB).

### Statistical Analyses

Statistical analyses were performed using SPSS for Windows version 22.0 (IBM Corp., Armonk, NY). Data on quantitative variables are presented as mean ± standard deviation and qualitative variables as numbers (percentage). The Chi-squared test was used for categorical variables. The independent samples *t*-test was used to compare continuous variables. Linear correlation was performed to assess the association between thyroid-related hormone hearing loss and hearing recovery. ROC curve analysis was used to assess the relationship between thyroid-related hormone and the occurrence of moderately severe-to-profound SSNHL. Binary logistic regression models were used to estimate the odds ratios (OR) and 95% confidence intervals (CI) for the correlation between thyroid-related hormone and the occurrence and outcome of moderately severe-to-profound SSNHL. The collinearity of all continuous variables was examined before performing the logistic regression using the variance inflation factor. *P* < 0.05 was considered significant for all tests. The figures were generated using GraphPad Prism 7.0 for Windows (GraphPad Software Inc., CA).

## Results

### Baseline and Clinical Characteristics of Participants

The baseline characteristics of participants are summarized in Table 1. About half of the patients (*n* = 38, 54.29%) were male and the mean age of the patients was 51.00 ± 15.89 years. There were no differences in age, sex distribution, height, weight, BMI, and blood pressure on admission between the two groups. From the results, the thyroid-related hormone levels of the moderately severe-to-profound SSNHL group were significantly lower than those of the control group (TT3, TT4, FT3, and TSH; all *P* < 0.05) as shown in Table 1. There is a known association between hypertension and diabetes with altered thyroid function (23, 24). We further compared the thyroid parameters of moderately severe-to-profound SSNHL with or without hypertension and diabetes. It was found that there were no significant differences in moderately severe-to-profound SSNHL subgroups (all P > 0.05) (as shown in Supplementary Tables 1, 2). This indicated that thyroid parameters between moderately severe-to-profound SSNHL group and control group may not be influenced by the clinical history of diabetes and hypertension.

**Table 1:** Baseline characteristics of participants in the moderately severe-to-profound SSNHL and control groups.

	**Moderately severe-to-profound SSNHL (*n* = 70)**	**Control (*n* = 100)**	***P*-value**
Baseline characteristics		
Age (years)	51.00 ± 15.89	49.73 ± 9.75	0.553
Sex (male, %)	38 (54.29)	51 (51.00)	0.673
Hight (cm)	167.49 ± 8.76	168.09 ± 7.52	0.631
Weight (kg)	68.25 ± 12.64	67.84 ± 11.39	0.826
BMI (kg/m2)	24.27 ± 3.69	23.92 ± 3.14	0.519
Systolic blood pressure (mmHg)	120.71 ± 16.93	123.69 ± 10.66	0.196
Diastolic blood pressure (mmHg)	76.01 ± 9.98	77.18 ± 9.73	0.448
Laboratory variables		
TT3 level (nmol/L)	1.27 ± 0.21	1.43 ± 0.20	<0.001^*^
TT4 level (nmol/L)	88.36 ± 14.56	96.67 ± 16.67	0.001^*^
FT3 level (pmol/L)	3.74 ± 0.64	4.58 ± 0.58	<0.001^*^
FT4 level (pmol/L)	16.41 ± 2.45	16.00 ± 2.15	0.249
TSH level (mIU/L)	1.46 ± 0.79	6.04 ± 4.87	<0.001^*^

*Data are expressed as a number with percentage for qualitative variables or mean ± standard deviation for quantitative variables. BMI, body mass index; TT3, total triiodothyronine; TT4, total thyroxine; FT3, free triiodothyronine; FT4, free thyroxine; TSH, thyroid stimulating hormone*.

* *The correlation was significant at the 0.05 level (P < 0.05)*.

Patients that experienced hearing recovery were divided into effective group (*n* = 30) and ineffective group (*n* = 40). As shown in Table 2, the baseline characteristics of the two groups had no significant differences (all *P* > 0.05). No significant differences were found between the two groups in terms of the affected side, accompanying symptoms such as tinnitus and ear fullness, and history of hypertension and diabetes (all *P* > 0.05). However, the number of patients with vertigo in the ineffective group was significantly higher than that in the effective group (*P* < 0.05). The time to treatment and the hearing loss level of the ineffective group were 4.95 ± 1.54 days and 82.11 ± 12.50 dBHL, respectively, and both were significantly higher than that in the effective group (3.57 ± 1.98 days and 75.43 ± 12.40 dBHL) (*P* < 0.05). There were no significant differences in thyroid-related hormone levels (all *P* > 0.05), however, changes in TT3, TT4, FT3, and TSH levels were significantly higher in the effective group than in the ineffective group (all *P* < 0.05).

**Table 2:** Demographics and laboratory variables in the moderately severe-to-profound SSNHL with different outcomes.

	**Effective**	**Ineffective**	***P*-value**
	**(*n* = 30)**	**(*n* = 40)**	
Baseline characteristics			
Age (years)	50.10 ± 16.97	51.68 ± 15.22	0.685
Sex (male, %)	15 (50.00)	23 (57.50)	0.533
Hight (cm)	168.13 ± 9.27	167.00 ± 8.45	0.596
Weight (kg)	69.12 ± 13.59	67.60 ± 12.00	0.629
BMI (kg/m2)	24.36 ± 3.73	24.20 ± 3.71	0.860
Systolic blood pressure (mmHg)	121.70 ± 16.15	119.98 ± 17.65	0.676
Diastolic blood pressure (mmHg)	77.07 ± 9.66	75.23 ± 10.26	0.445
Clinical characteristics			
Affected side (left, %)	20 (66.67)	19 (47.50)	0.110
Tinnitus (%)	13 (43.33)	18 (45.00)	0.890
Vertigo (%)	3 (10.00)	15 (37.50)	0.009^*^
Ear fullness (%)	3 (10.00)	6 (15.00)	0.536
Hypertension (%)	4 (13.33)	8 (20.00)	0.464
Diabetes (%)	4 (13.33)	6 (15.00)	0.844
Time to treatment (days)	3.57 ± 1.98	4.95 ± 1.54	0.002^*^
Hearing level (dBHL)	75.43 ± 12.40	82.11 ± 12.50	0.030^*^
Laboratory variables			
TT3 level (nmol/L)	1.26 ± 0.19	1.28 ± 0.22	0.697
TT4 level (nmol/L)	89.08 ± 14.21	87.81 ± 14.98	0.722
FT3 level (pmol/L)	3.66 ± 0.60	3.79 ± 0.67	0.393
FT4 level (pmol/L)	16.48 ± 2.75	16.36 ± 2.22	0.844
TSH level (mIU/L)	1.50 ± 0.74	1.42 ± 0.83	0.653
ΔTT3 level (nmol/L)	0.29 ± 0.16	0.10 ± 0.39	0.006^*^
ΔTT4 level (nmol/L)	15.21 ± 18.85	5.71 ± 19.87	0.047^*^
ΔFT3 level (pmol/L)	1.00 ± 0.58	0.40 ± 0.94	0.002^*^
ΔFT4 level (pmol/L)	0.35 ± 3.13	−0.65 ± 2.93	0.174
ΔTSH level (mIU/L)	4.94 ± 3.21	1.92 ± 1.62	<0.001^*^

*Data are expressed as a number with percentage for qualitative variables or mean ± standard deviation for quantitative variables. BMI, body mass index; TT3, total triiodothyronine; TT4, total thyroxine; FT3, free triiodothyronine; FT4, free thyroxine; TSH, thyroid stimulating hormone; Δ, the difference of thyroid-related hormone after treatment minus that before treatment*.

* *The correlation was significant at the 0.05 level (P < 0.05)*.

### Thyroid-Related Hormone and the Occurrence of Moderately Severe-to-Profound SSNHL

**Figure 1:**
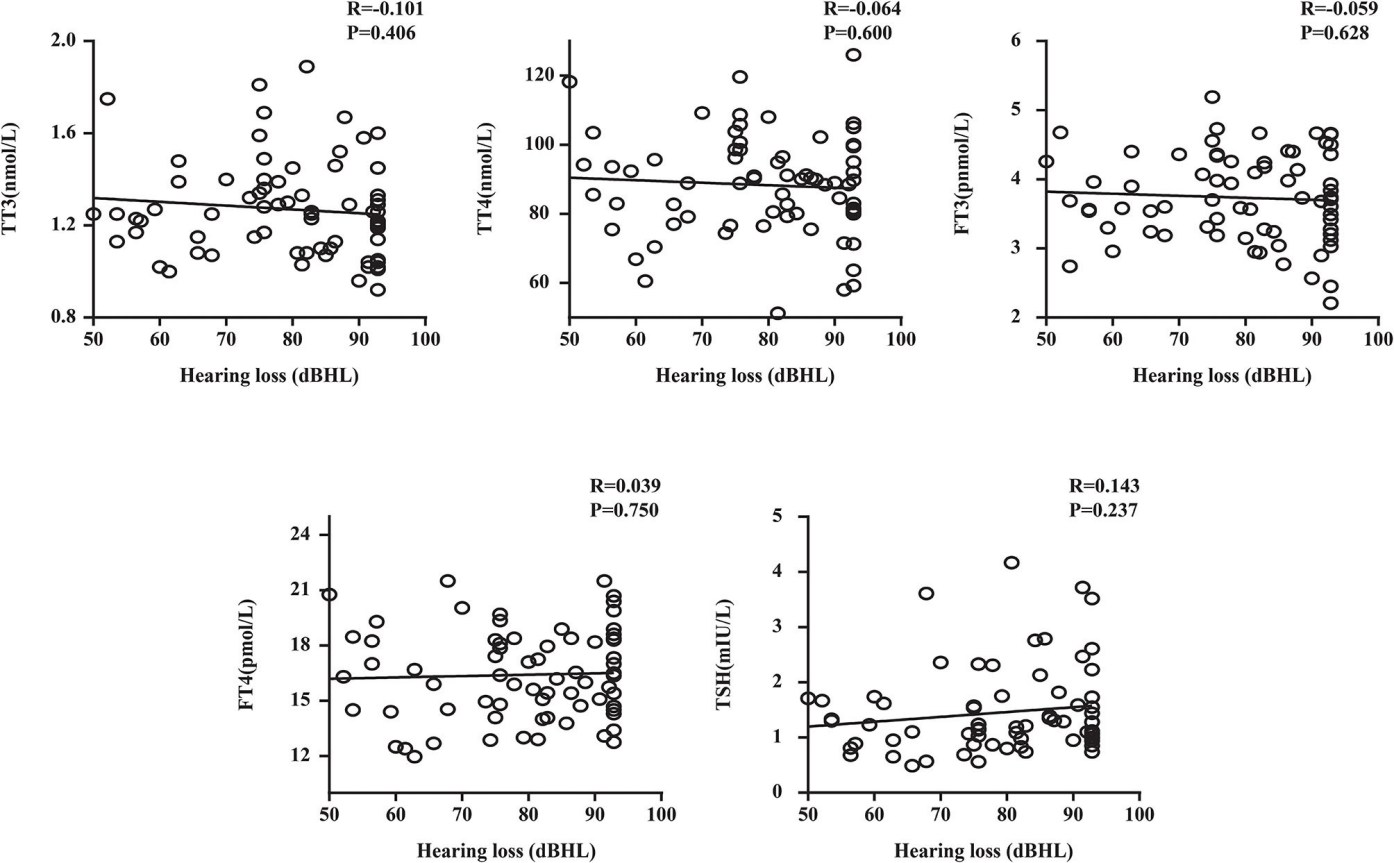
Plots of thyroid-related hormone levels vs. the severity of hearing loss. TT3, total triiodothyronine; TT4, total thyroxine; FT3, free triiodothyronine; FT4, free thyroxine; TSH, thyroid stimulating hormone; R, correlation coefficient. Data are presented as correlation coefficients and *P*-values.

**Figure 2:**
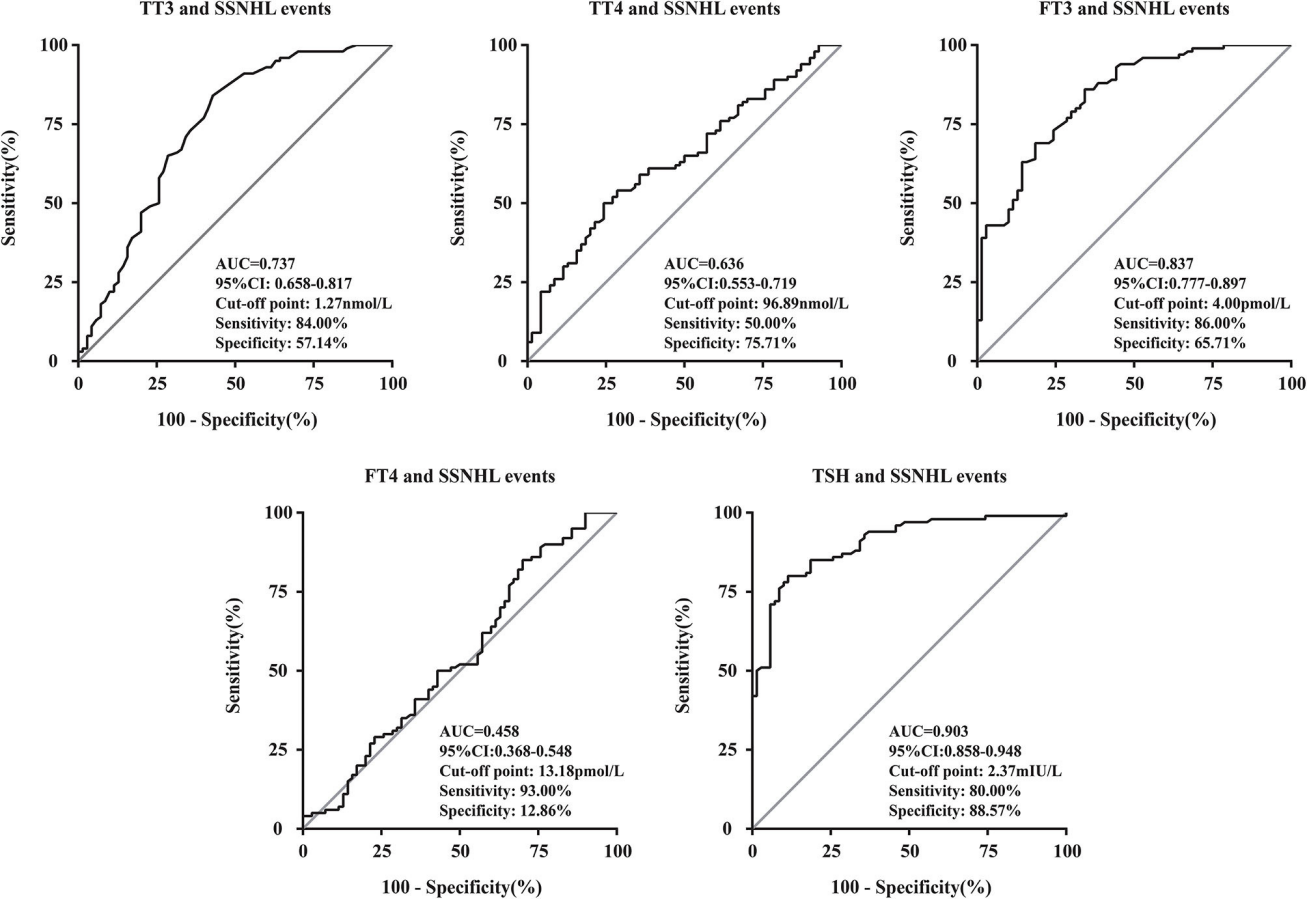
Receiver operating characteristic curve analysis of thyroid-related hormone levels for the prediction of the occurrence of moderately severe-to-profound SSNHL. AUC, area under the curve; TT3, total triiodothyronine; TT4, total thyroxine; FT3, free triiodothyronine; FT4, free thyroxine; TSH, thyroid stimulating hormone; CI, confidence interval; SSNHL, sudden sensorineural hearing loss.

We performed a linear correlation analysis and drew the scatterplots of thyroid-related hormone levels vs. severity of hearing loss (Figure 1) and found no association between moderately severe-to-profound SSNHL and TSH when hearing loss was treated as a continuous variable. The ROC curve analysis (Figure 2) revealed that TT3 level ≤ 1.27 nmol/L (sensitivity, 84.00%; specificity, 57.14%), TT4 level ≤ 96.89 nmol/L (sensitivity, 50.001%; specificity, 75.71%), FT3 level ≤ 4.00 pmol/L (sensitivity, 86.00%; specificity, 65.71%), FT4 level ≤ 13.18 pmol/L (sensitivity, 93.00%; specificity, 12.86%), and TSH level ≤ 2.37 mIU/L (sensitivity, 80.00%; specificity, 88.57%) were the most powerful predictors of moderately severe-to-profound SSNHL. The areas under the curve were 0.737 (95% CI, 0.658–0.817), 0.636 (95% CI, 0.553–0.719), 0.837 (95% CI, 0.777–0.897), 0.458 (95% CI, 0.368–0.548), and 0.903 (95% CI, 0.858–0.948) for TT3, TT4, FT3, FT4, and TSH, respectively. FT3 and TSH levels showed good predictive efficacies for the occurrence of moderately severe-to-profound SSNHL. In the univariate logistic regression analysis, the ORs for occurrence outcome of disease with parameters are presented in Table 3. With unadjusted ORs of 0.093 (95% CI, 0.044–0.195, *P* < 0.05) and 0.245 (95% CI, 0.155–0.389, *P* < 0.05), FT3 and TSH levels showed a strong association with the occurrence of moderately severe-to-profound SSNHL. After adjusting for all other significant predictors, FT3 and TSH levels remained independent occurrence predictors with adjusted ORs of 0.064 (95% CI, 0.016–0.255, *P* < 0.05) and 0.270 (95% CI, 0.157–0.462, *P* < 0.05).

**Table 3:** Binary logistic regression analysis of the relationship between thyroid-related hormone levels and the occurrence of moderately severe-to-profound SSNHL.

**Parameter**	**Univariate analysis**			**Multivariate analysis**		
	**OR**	**95% CI**	***P*-value**	**OR**	**95% CI**	***P*-value**
Predictor: occurrence of disease						
Age	1.008	0.984–1.033	0.518			
Sex	1.142	0.618–2.105	0.673			
BMI	1.031	0.941–1.129	0.517			
Systolic blood pressure	0.984	0.962–1.007	0.162			
Diastolic blood pressure	0.988	0.958–1.019	0.446			
TT3 level	0.133	0.020–0.892	<0.001^*^	5.262	0.174–158.842	0.339
TT4 level	0.966	0.946–0.987	0.002^*^	0.974	0.934–1.015	0.211
FT3 level	0.093	0.044–0.195	<0.001^*^	0.064	0.016–0.255	<0.001^*^
FT4 level	1.083	0.946–1.241	0.248			
TSH level	0.245	0.155–0.389	<0.001^*^	0.270	0.157–0.462	<0.001^*^

*OR, odds ratio; CI, confidence interval; BMI, body mass index; TT3, total triiodothyronine; TT4, total thyroxine; FT3, free triiodothyronine; FT4, free thyroxine; TSH, thyroid stimulating hormone*.

* *The correlation was significant at the 0.05 level (P < 0.05)*.

### Thyroid-Related Hormone and the Functional Outcome of Moderately Severe-to-Profound SSNHL

**Figure 3:**
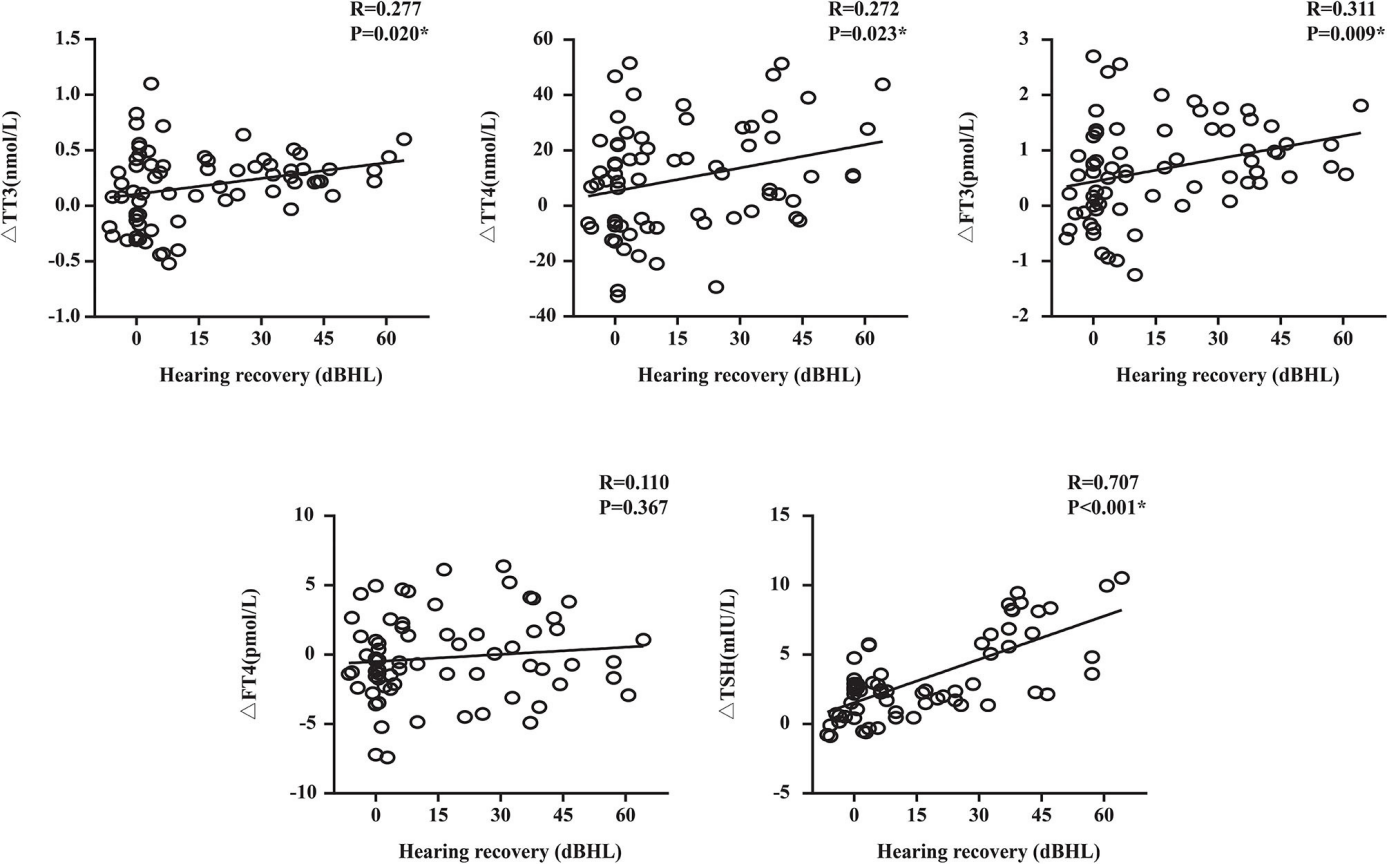
Plots of the change in thyroid-related hormone levels against the recovery of hearing. TT3, total triiodothyronine; TT4, total thyroxine; FT3, free triiodothyronine; FT4, free thyroxine; TSH, thyroid stimulating hormone; R, correlation coefficient; Δ, the difference of thyroid-related hormone after treatment minus that before treatment. Data are presented as correlation coefficients and *P*-values. *The correlation was significant at the 0.05 level (*P* < 0.05). Hearing recovery was positively correlated with the elevation of TSH levels (*R* = 0.707, *P* < 0.05).

In the linear correlation analysis and scatterplots of the elevation of thyroid-related hormone levels vs. hearing recovery (Figure 3), an association was found between moderately severe-to-profound SSNHL and TSH when hearing loss was treated as a continuous variable. The elevation of TSH level (*R* = 0.707, *P* < 0.05) after treatment predicted a favorable outcome of hearing recovery. In the univariate logistic regression analysis, the ORs for the outcome of moderately severe-to-profound SSNHL with parameters are presented in Table 4. We found parameters that include vertigo, time to treatment, hearing level, and the change in TT3, FT3, and TSH levels to show strong associations with the treatment outcome. Given that cardiovascular risk factors such as hypertension and diabetes may be associated with SSNHL, we performed a multivariate logistic regression model adjusted using these two factors along with those that were significant in the univariate analysis. Using the variance inflation factor, all parameters were deemed non-collinear and could be used in further analysis. It was found that the change in TSH level remained the independent outcome predictor with an adjusted OR of 1.456 (95% CI, 1.069–1.983, *P* < 0.05).

**Table 4:** Binary logistic regression analysis of the relationship between thyroid-related hormone levels and the outcome of moderately severe-to-profound SSNHL.

**Parameter**	**Univariate analysis**			**Multivariate analysis**		
	**OR**	**95% CI**	***P*-value**	**OR**	**95% CI**	***P*-value**
Predictor: outcome of disease						
Age	0.994	0.964–1.024	0.680			
Sex (male)	0.739	0.285–1.914	0.533			
BMI	1.012	0.889–1.151	0.858			
Systolic blood pressure	1.006	0.978–1.035	0.671			
Diastolic blood pressure	1.019	0.971–1.070	0.443			
Affected side (left)	2.211	0.829–5.893	0.113			
Tinnitus	1.070	0.412–2.777	0.890			
Vertigo	5.400	1.395–20.907	0.015^*^	3.378	0.677–16.855	0.138
Ear fullness	1.588	0.363–6.943	0.539			
Hypertension	1.625	0.440–6.005	0.467	2.449	0.401–14.956	0.332
Diabetes	1.147	0.293–4.488	0.844	1.837	0.241–14.032	0.558
Time to treatment	0.641	0.477–0.861	0.003^*^	0.697	0.478–1.015	0.060
Hearing level	0.958	0.921–0.997	0.034^*^	0.956	0.906–1.010	0.107
TT3 level	0.629	0.063–6.253	0.692			
TT4 level	1.006	0.974–1.040	0.717			
FT3 level	0.717	0.336–1.527	0.388			
FT4 level	1.021	0.840–1.241	0.836			
TSH level	1.151	0.630–2.103	0.648			
ΔTT3 level	7.605	1.428–40.504	0.017^*^	1.120	0.049–25.606	0.943
ΔTT4 level	1.026	1.000–1.053	0.052			
ΔFT3 level	2.533	1.300–4.937	0.006^*^	2.048	0.583–7.194	0.263
ΔFT4 level	1.119	0.951–1.317	0.175			
ΔTSH level	1.656	1.267–2.165	<0.001^*^	1.456	1.069–1.983	0.017^*^

*OR, odds ratio; CI, confidence interval; BMI, body mass index; TT3, total triiodothyronine; TT4, total thyroxine; FT3, free triiodothyronine; FT4, free thyroxine; TSH, thyroid stimulating hormone*.

* *The correlation was significant at the 0.05 level (P < 0.05)*.

## Discussion

SSNHL is a common otological emergency with an increasing incidence rate worldwide. It can cause long-term hearing loss, irreversible hearing loss, and tinnitus (25, 26), severely affecting patients' quality of life. Recently, many studies have found that hematological indices, such as routine blood parameters, serum lipid level, and coagulation function are convenient indicators for prognosis in patients with SSNHL (26–28). A large, representative population cohort studied by Kim et al. (29) showed that SSNHL patients are more likely to have goiter and hypothyroidism than normal people. In addition, a large case-control study in Taiwan reported that pre-existing hypothyroidism and hyperthyroidism are associated with SSNHL susceptibility (30). This study is the first to show an association between thyroid-related hormone before and after treatment in patients with moderately severe-to-profound SSNHL.

### Lower Thyroid-Related Hormone Levels Reflect Higher Risk of Moderately Severe-to-Profound SSNHL

In this study, although still within normal ranges, we found that the levels of thyroid-related hormones were significantly lower in patients with moderately severe-to-profound SSNHL than those of the control group (Table 1). Analyzing the relationship between thyroid-related hormones and SSNHL through ROC curve suggested that the AUC for FT3 diagnosis of SSNHL is 0.837, with a cut-off point at 4.00 pmol/L (sensitivity, 86.00%; specificity, 65.71%). In terms of TSH, diagnosis for SSNHL is 0.903, with a cut-off point at 2.37 mIU/L (sensitivity, 80.00%; specificity, 88.57%). This shows that early detection of FT3 and TSH are valuable for the diagnosis of SSNHL (Figure 2). Lower FT3 and TSH levels in the early stages of moderately severe-to-profound SSNHL were independent predictors of the occurrence of moderately severe-to-profound SSNHL (Table 3).

### Ischemia and Lower Neuroprotection Contribute to the Higher Risk of Moderately Severe-to-Profound SSNHL

The mechanisms behind our findings may be understood from several established research. Hypothyroidism is related to the severity of atherosclerosis risk factors (hypertension, hyperlipemia, hyperhomocysteinemia) and may be a risk factor of atherothrombotic myocardial infarction (15). Low triiodothyronine (T3) level also plays a role in vascular diseases, including complications after brain tumor surgery, respiratory failure, and acute cardiovascular events (31–33). Given that the blood supply of the inner ear is mainly dependent on end arterioles, the function of the inner ear is greatly affected by ischemia, and ischemia may lead to hearing loss at all frequencies when thrombosis occurs in the peripheral arterioles (34).

In regard to the association of poor outcomes of moderately severe-to-profound SSNHL with low serum thyroid hormone levels in patients, one possible mechanism is secondary neuronal damage after moderately severe-to-profound SSNHL. Wang et al. (35) reported that a decrease in TSH levels and an increase in the basal metabolic rate may lead to a higher risk of post-stroke fatigue; this could lead to the production of excess reactive oxygen species and free radicals, resulting in neurotoxicity (36). In addition, thyroid-related hormones and their derivatives have a great influence on the repair of injured neurons (37, 38). Sadana et al. (39) reported that T3 can reduce infarct and edema in a focal ischemia model. Therefore, we speculate that patients with hypothyroidism may experience lower neuroprotection and worsened secondary damage, leading to a poor outcome.

Furthermore, a growing body of research found that thyroid-related hormone levels are greatly associated with the secretion of many neurotrophic factors, including nerve growth factors (40, 41). Therefore, we can infer that patients with hypothyroidism may experience inhibition of endogenous neuron repair systems, leading to poorer functional outcomes. The elevation of TSH levels after treatment predicts a favorable outcome of hearing recovery (Figure 3), evident from the greater increase in TT3, TT4, FT3, and TSH levels for the effective group than that in the ineffective group (Table 2), while TSH level remained an independent outcome predictor (Table 4). Tamura et al. (42) found that steroid can not only improve thyroid function but also lead to transient decrease in thyroid hormone and an increase in reverse thyroid hormone. Most patients presented an upward trend of thyroid-related hormone levels after glucocorticoid treatment. This may explain why some patients had thyroid hormone increase after glucocorticoid treatment and thus a better prognosis.

Our study has some limitations. First, being a preliminary study, the repeatability of the results needs to be verified in more prospective studies to determine their stability and effectiveness. Second, although the thyroid hormones were correlated with hypertension and diabetes in previous reports (23, 24), this correlation was not significant in our study. This could be due to the small sample size, where the association between thyroid-related hormone levels and cardiovascular status could not be identified. Third, the study has not proven a causal relationship between moderately severe-to-profound SSNHL and thyroid-related hormone levels, thus a randomized controlled trial is needed for further confirmation.

## Conclusions

In summary, our study indicated that thyroid-related hormones play an important role in the clinical characteristics and outcome of moderately severe-to-profound SSNHL. During in-hospital monitoring, thyroid function testing may be important as higher levels of thyroid-related hormones have been associated with better functional outcomes. Therefore, treatment to normalize and/or elevate thyroid-related hormone levels among patients with moderately severe-to-profound SSNHL could be beneficial to their recovery. Further prospective studies with longer-term follow-up are needed to confirm our findings.

## Data Availability Statement

The raw data supporting the conclusions of this article will be made available by the authors, without undue reservation.

## Ethics Statement

The studies involving human participants were reviewed and approved by Shanghai Jiaotong University Affiliated Sixth People' s Hospital Ethics Committee. The patients/participants provided their written informed consent to participate in this study.

## Author Contributions

ZZ, YiS, YF, and SY: conceptualization. ZZ, LiaX, LilX, and YuS: data curation. YiS: formal analysis. YiS, LiaX, LilX, YuS, and JH: investigation. YF and SY: project administration and writing—review and editing. HW, ZC, YW, HS, and YF: resources. HW, ZC, YW, HS, and SY: supervision. JH: validation. ZZ: writing—original draft. All authors contributed to the article and approved the submitted version.

## Funding

This study was supported by the National Natural Science Foundation of China (81771015); the First Grant (2020YFC2005201) of Chinese National Key Research and Development Program (2020YFC2005200); and Shanghai Municipal Commission of Science and Technology (Grant No.18DZ2260200).

## Conflict of Interest

The authors declare that the research was conducted in the absence of any commercial or financial relationships that could be construed as a potential conflict of interest.

## Publisher's Note

All claims expressed in this article are solely those of the authors and do not necessarily represent those of their affiliated organizations, or those of the publisher, the editors and the reviewers. Any product that may be evaluated in this article, or claim that may be made by its manufacturer, is not guaranteed or endorsed by the publisher.

## References

[1] Chandrasekhar SS Tsai Do BS Schwartz SR Bontempo LJ Faucett EA Finestone SA. Clinical practice guideline: sudden hearing loss (update). Otolaryngology. (2019) 161(1_suppl):S1–45. 10.1177/0194599819859885 31369359

[2] Mattox DE Simmons FB. Natural history of sudden sensorineural hearing loss. Ann Otol Rhinol Laryngol. (1977) 86(4 Pt 1):463–80. 10.1177/000348947708600406 889223

[3] Byl FM. Seventy-six cases of presumed sudden hearing loss occurring in 1973: prognosis and incidence. Laryngoscope. (1977) 87 (5 Pt 1):817–25. 10.1002/lary.5540870515 850455

[4] Alexander TH Harris JP. Incidence of sudden sensorineural hearing loss. Otol Neurotol. (2013) 34:1586–9. 10.1097/MAO.0000000000000222 24232060

[5] Schreiber BE Agrup C Haskard DO Luxon LM. Sudden sensorineural hearing loss. Lancet. (2010) 375:1203–11. 10.1016/S0140-6736(09)62071-7 20362815

[6] Editorial Board of Chinese Journal of Otorhinolaryngology Head and Neck Surgery. [Guideline of diagnosis and treatment of sudden deafness (2015)]. Zhonghua Er Bi Yan Hou Tou Jing Wai Ke Za Zhi. (2015) 50:443–7. 26695792

[7] Li BH Jiang ZG. [Curative effect analysis of different degree of hearing lossin sudden deafness patients]. Lin Chung Er Bi Yan Hou Tou Jing Wai Ke Za Zhi. (2016) 30:1124–6. 10.13201/j.issn.1001-1781.2016.14.008 29798436

[8] Sheehy JL. Vasodilator therapy in sensory-neural hearing loss. Laryngoscope. (1960) 70:885–914 10.1288/00005537-196007000-00002 14445724

[9] Enache R Sarafoleanu C Prognostic factors in sudden hearing loss. J Med Life. (2008) 1:343–7. 20108511 PMC3018969

[10] Wilson WR Byl FM Laird N. The efficacy of steroids in the treatment of idiopathic sudden hearing loss. A double-blind clinical study. Arch Otolaryngol. (1980) 106:772–6 10.1001/archotol.1980.00790360050013 7002129

[11] WHO. World Report on Hearing (2021). Available online at: https://www.whoint/publications/i/item/world-report-on-hearing Available online at: (accessed March 03, 2021).

[12] Yang MH Yang FY Lee DD. Thyroid disease as a risk factor for cerebrovascular disease. J Stroke Cerebrovasc Dis. (2015) 24:912–20. 10.1016/j.jstrokecerebrovasdis.2014.11.032 25804562

[13] Forti P Maioli F Coveri M Nativio V Arnone G Loreti A. Thyroid function tests and early outcomes of acute ischemic stroke in older euthyroid patients. Exp Gerontol. (2015) 61:8–14. 10.1016/j.exger.2014.11.011 25449856

[14] Martin SS Daya N Lutsey PL Matsushita K Fretz A McEvoy JW. Thyroid function, cardiovascular risk factors, and incident atherosclerotic cardiovascular disease: the Atherosclerosis Risk in Communities (ARIC) study. J Clin Endocrinol Metab. (2017) 102:3306–15. 10.1210/jc.2017-00986 28605456 PMC5587060

[15] Squizzato A Gerdes VE Brandjes DP Buller HR Stam J. Thyroid diseases and cerebrovascular disease. Stroke. (2005) 36:2302–10. 10.1161/01.STR.0000181772.78492.07 16179578

[16] Soriguer F Millon MC Munoz R Mancha I Lopez Siguero JP Martinez Aedo MJ. The auditory threshold in a school-age population is related to iodine intake and thyroid function. Thyroid. (2000) 10:991–9. 10.1089/thy.2000.10.991 11128728

[17] Everett LA Glaser B Beck JC Idol JR Buchs A Heyman M. Pendred syndrome is caused by mutations in a putative sulphate transporter gene (PDS). Nat Genet. (1997) 17:411–22. 10.1038/ng1297-411 9398842

[18] Ng L Cordas E Wu X Vella KR Hollenberg AN Forrest D Age-related hearing loss and degeneration of cochlear hair cells in mice lacking thyroid hormone receptor beta1. Endocrinology (2015) 156:3853–65. 10.1210/en.2015-1468 26241124 PMC4588828

[19] Forrest D Erway LC Ng L Altschuler R Curran T. Thyroid hormone receptor beta is essential for development of auditory function. Nat Genet. (1996) 13:354–7. 10.1038/ng0796-354 8673137

[20] Richter CP Munscher A Machado DS Wondisford FE Ortiga-Carvalho TM. Complete activation of thyroid hormone receptor beta by T3 is essential for normal cochlear function and morphology in mice. Cell Physiol Biochem. (2011) 28:997–1008. 10.1159/000335812 22178950 PMC3709180

[21] Ng L Goodyear RJ Woods CA Schneider MJ Diamond E Richardson GP. Hearing loss and retarded cochlear development in mice lacking type 2 iodothyronine deiodinase. Proc Natl Acad Sci USA. (2004) 101:3474–9. 10.1073/pnas.0307402101 14993610 PMC373486

[22] Li D Henley CM O'Malley BW Jr. Distortion product otoacoustic emissions and outer hair cell defects in the hyt/hyt mutant mouse. Hearing Res. (1999) 138:65–72. 10.1016/S0378-5955(99)00150-1 10575115

[23] Cai P Peng Y Chen Y Li L Chu W Wang Y. Association of thyroid function with white coat hypertension and sustained hypertension. J Clin Hypertension. (2019) 21:674–83. 10.1111/jch.13536 30973206 PMC8030441

[24] Reismann P Somogyi A. Diabetes and thyroid disorders. Orvosi Hetilap. (2011) 152:516–9. 10.1556/OH.2011.29056 21398213

[25] Heigl F Hettich R Suckfuell M Luebbers CW Osterkorn D Osterkorn K. Fibrinogen/LDL apheresis as successful second-line treatment of sudden hearing loss: a retrospective study on 217 patients. Atheroscler Suppl. (2009) 10:95–101. 10.1016/S1567-5688(09)71820-3 20129384

[26] Cho SH Chen H Kim IS Yokose C Kang J Cho D. Association of the 4 g/5 g polymorphism of plasminogen activator inhibitor-1 gene with sudden sensorineural hearing loss. A case control study. BMC Ear Nose Throat Disord. (2012) 12:5 10.1186/1472-6815-12-5 22672326 PMC3431267

[27] Weng T Devine EE Xu H Yan Z Dong P. A clinical study of serum lipid disturbance in Chinese patients with sudden deafness. Lipids Health Dis. (2013) 12:95. 10.1186/1476-511X-12-95 23819577 PMC3716951

[28] Bao F Zhang S Zhang Y Zhu X Liu W. The correlation analysis of coagulation detection and blood routine parameters of sudden hearing loss. J Clin Otorhinolaryngol Head Neck Surg. (2015) 29:52–6. 25966556

[29] Kim SY Song YS Wee JH Min C Yoo DM Choi HG. Association between SSNHL and Thyroid Diseases. Int J Environ Res Public Health. (2020) 17:8419. 10.3390/ijerph17228419 33202999 PMC7697232

[30] Tsai YT Chang IJ Hsu CM Yang YH Liu CY Tsai MS. Association between sudden sensorineural hearing loss and preexisting thyroid diseases: a nationwide case-control study in Taiwan. Int J Environ Res Public Health. (2020) 17:834. 10.3390/ijerph17030834 32013113 PMC7037331

[31] Bunevicius A Deltuva V Tamasauskas S Tamasauskas A Laws ER Jr Bunevicius R. Low triiodothyronine syndrome as a predictor of poor outcomes in patients undergoing brain tumor surgery: a pilot study: clinical article. J Neurosurg. (2013) 118:1279–87. 10.3171/2013.1.JNS121696 23480214

[32] Scoscia E Baglioni S Eslami A Iervasi G Monti S Todisco T. Low triiodothyronine (T3) state: a predictor of outcome in respiratory failure? Results of a clinical pilot study. Eur J Endocrinol. (2004) 151:557–60. 10.1530/eje.0.1510557 15538932

[33] Iervasi G Pingitore A Landi P Raciti M Ripoli A Scarlattini M. Low-T3 syndrome: a strong prognostic predictor of death in patients with heart disease. Circulation. (2003) 107:708–13. 10.1161/01.CIR.0000048124.64204.3F 12578873

[34] Rauch SD. Clinical practice. Idiopathic sudden sensorineural hearing loss. N Engl J Med. (2008) 359:833–40. 10.1056/NEJMcp0802129 18716300

[35] Wang J Li F Xiao L Peng F Sun W Li M. Depressed TSH level as a predictor of poststroke fatigue in patients with acute ischemic stroke. Neurology. (2018) 91:e1971–8. 10.1212/WNL.0000000000006534 30366976

[36] Aslan M Cosar N Celik H Aksoy N Dulger AC Begenik H. Evaluation of oxidative status in patients with hyperthyroidism. Endocrine. (2011) 40:285–9. 10.1007/s12020-011-9472-3 21519910

[37] Mendes-de-Aguiar CB Alchini R Decker H Alvarez-Silva M Tasca CI Trentin AG. Thyroid hormone increases astrocytic glutamate uptake and protects astrocytes and neurons against glutamate toxicity. J Neurosci Res. (2008) 86:3117–25. 10.1002/jnr.21755 18543341

[38] Kaasik A Minajeva A Paju K Eimre M Seppet EK. Thyroid hormones differentially affect sarcoplasmic reticulum function in rat atria and ventricles. Mol Cell Biochem. (1997) 176:119–26. 10.1023/A:1006887231150 9406153

[39] Sadana P Coughlin L Burke J Woods R Mdzinarishvili A. Anti-edema action of thyroid hormone in MCAO model of ischemic brain stroke: possible association with AQP4 modulation. J Neurol Sci. (2015) 354:37–45. 10.1016/j.jns.2015.04.042 25963308

[40] Sui L Ren WW Li BM. Administration of thyroid hormone increases reelin and brain-derived neurotrophic factor expression in rat hippocampus *in vivo*. Brain Res. (2010) 1313:9–24. 10.1016/j.brainres.2009.12.010 20018181

[41] Kolb B Cote S Ribeiro-da-Silva A Cuello AC. Nerve growth factor treatment prevents dendritic atrophy and promotes recovery of function after cortical injury. Neuroscience. (1997) 76:1139–51. 10.1016/S0306-4522(96)00448-4 9027874

[42] Tamura M Yokoyama N Nishikawa T Takeshita A Kimura H Ashizawa K. Improvement of hypothyroidism after glucocorticoid replacement in isolated adrenocorticotropin deficiency. Intern Med. (1995) 34:559–63. 10.2169/internalmedicine.34.559 7549143

