# Supplementary material for: Thyroid-Related Hormone Levels in Clinical Patients With Moderately Severe-to-Profound Sudden Sensorineural Hearing Loss: A Prospective Study
Source: Front Neurol. 2021 Oct 28;12:753270. doi: 10.3389/fneur.2021.753270 (PMC8581239; doi:10.3389/fneur.2021.753270)
Supplement: Supplementary file 1 [file Table_1.DOCX]

**Supplementary materials**

Supplementary Table 1. Laboratory variables of moderately severe-to-profound SSNHL with or without hypertension

|  | Hypertensive patients (n=12) | Non hypertensive patients (n=58) | P value |
| --- | --- | --- | --- |
| *TT3 level (nmol/L)* | 1.27±0.16 | 1.27±0.22 | 0.998 |
| *TT4 level (nmol/L)* | 93.07±13.16 | 87.38±14.75 | 0.220 |
| *FT3 level (pmol/L)* | 3.66±0.56 | 3.75±0.66 | 0.640 |
| *FT4 level (pmol/L)* | 17.00±1.89 | 16.29±2.54 | 0.363 |
| *TSH level (mIU/L)* | 1.33±0.94 | 1.48±0.76 | 0.562 |

Supplementary Table 2. Laboratory variables of moderately severe-to-profound SSNHL with or without diabetes

|  | Diabetic patients (n=10) | Nondiabetic patients (n=60) | P value |
| --- | --- | --- | --- |
| *TT3 level (nmol/L)* | 1.27±0.20 | 1.27±0.21 | 0.956 |
| *TT4 level (nmol/L)* | 92.84±13.51 | 87.61±14.70 | 0.296 |
| *FT3 level (pmol/L)* | 3.49±0.70 | 3.78±0.62 | 0.181 |
| *FT4 level (pmol/L)* | 16.74±2.46 | 16.36±2.46 | 0.651 |
| *TSH level (mIU/L)* | 1.17±0.46 | 1.50±0.82 | 0.221 |
